# Use of Angiotensin Receptor-Neprilysin Inhibitors (ARNis) in High-Output Heart Failure in a Young Hemodialysis Patient: Synergistic or Cumulative Effect?

**DOI:** 10.7759/cureus.99973

**Published:** 2025-12-23

**Authors:** Sara R Silva, Elsa Soares, Ana Natário, Artur Gama, Teresa Bernardo

**Affiliations:** 1 Internal Medicine, Hospital do Litoral Alentejano, Santiago do Cacém, PRT; 2 Nephrology, Centro Hospitalar De Setúbal, Setúbal, PRT; 3 Nephrology, Unidade Local de Saúde da Arrábida, Setúbal, PRT

**Keywords:** chronic kidney disease, hemodialysis, high-flow arteriovenous fistula, high-output heart failure, hypertension, sacubitril/valsartan

## Abstract

In hemodialysis patients, an arteriovenous fistula (AVF) is the preferred option for vascular access. High-output heart failure (HOHF) due to a high-flow AVF can be an underrecognized complication, and arterial hypertension is among the most prevalent comorbidities in this population. Consequently, these patients are closely monitored during hemodialysis and routinely evaluated for cardiovascular pathology. A 32-year-old woman on hemodialysis for five years presented with hypertension refractory to triple pharmacologic therapy and dialysis, pleural effusion, exertional dyspnea, peripheral edema, and jugular venous distention. She was evaluated at an internal medicine day hospital to investigate the underlying cause of recurrent pleural effusion. She was found to have a concomitant high-flow AVF (Qa >3000 mL/min), which had undergone multiple banding procedures. Thoracentesis with cytobiochemical analysis revealed a transudative pleural effusion. Transthoracic echocardiography (TTE) demonstrated HF with reduced ejection fraction (38%) and pulmonary artery systolic pressure (PASP) of 46 mmHg; N-terminal pro-BNP (NT-proBNP) was 113,883 pg/mL. After multidisciplinary discussion, all antihypertensive therapy was replaced with intermediate-dose sacubitril/valsartan (SAC/VAL), aiming to control blood pressure and improve ejection fraction, with close monitoring of serum potassium and hemodynamics. Within two weeks, blood pressure control and symptoms improved significantly, with resolution of the pleural effusion. Two months after initiating SAC/VAL, the AVF was surgically closed, and dialysis access was switched to a long-term catheter. At six-month follow-up, repeat TTE showed recovery of ejection fraction to 71% and reduction of PASP to 23 mmHg. NT-proBNP decreased to 2,005 pg/mL, corroborating clinical improvement. The patient remains clinically stable. Continuous clinical assessment and individualized management are essential for addressing complex conditions in dialysis patients. Further research is warranted to establish the efficacy and safety of SAC/VAL in this population.

## Introduction

Heart failure (HF) is a common and serious complication in patients undergoing hemodialysis, contributing significantly to morbidity and mortality [1]. High-output HF (HOHF), secondary to high-flow arteriovenous fistulas (AVFs), is an underrecognized condition in this population [1,2]. The association between HOHF and high-flow AVF has been described since the 1970s [1].

HOHF is defined by a cardiac index greater than 4 L/min/m² or an absolute cardiac output greater than 8 L/min, accompanied by clinical signs and symptoms of HF [1]. This contrasts with typical low-output HF, where reduced cardiac output is the primary issue. In high-output states, the fundamental hemodynamic abnormality is markedly reduced systemic vascular resistance (SVR), creating a depressurized circulatory system that obligates the heart to work harder to maintain organ perfusion [1]. The reduction in SVR leads to sympathetic activation and stimulation of the renin-angiotensin-aldosterone system, resulting in fluid retention, cardiac remodeling, and progressive cardiac chamber dilation [1].

Hypertension is highly prevalent among dialysis patients and represents an additional cardiovascular risk factor [3]. Consequently, hemodialysis patients undergo annual echocardiograms and regular AVF blood flow rate (Qa) measurements, even in younger individuals. HOHF is more frequent in patients with upper-limb AVFs and Qa >2000 mL/min [1]. Blood pressure is monitored routinely during hemodialysis and at home, with periodic assessments by the attending nephrologist.

HF management in hemodialysis patients follows standard HF classification; however, several cornerstone drugs require caution or are restricted due to impaired renal function and increased risk of adverse effects such as hyperkalemia [4]. This particularly applies to angiotensin receptor-neprilysin inhibitors (ARNis), angiotensin-converting enzyme (ACE) inhibitors, angiotensin receptor blockers (ARBs), and mineralocorticoid receptor antagonists. Sodium-glucose cotransporter-2 inhibitors lack robust safety data in this population [4]. Additionally, hemodialysis patients are typically excluded from major HF drug trials, limiting guideline-based recommendations for this group.

Available evidence suggests that, in hemodialysis patients with HF with reduced ejection fraction (HFrEF), treatment with a beta-blocker combined with an ACE inhibitor, ARB, or ARNi is preferable to no HFrEF-specific therapy or other pharmacologic regimens [4]. Management usually includes pharmacologic therapy, lifestyle modifications, optimization of comorbidities, and, when appropriate, device therapy [4]. Fluid overload requires careful handling depending on residual urine output, diuretic resistance, and the patient’s tolerance to higher ultrafiltration volumes during dialysis [4]. Optimization of dialysis parameters, including dialysate composition, ultrafiltration rate, and treatment frequency, is crucial for maintaining intradialytic hemodynamic stability and minimizing cardiac complications [4]. Other key aspects include management of anemia and systematic assessment for device-based therapies such as cardiac resynchronization therapy and implantable cardioverter-defibrillators [4].

Sacubitril/valsartan (SAC/VAL), an ARNi, represents an innovative therapeutic approach for managing HFrEF [5]. SAC/VAL may also be a promising treatment for hypertension in hemodialysis patients [3,6]. SAC inhibits neprilysin, which degrades vasoactive peptides such as natriuretic peptides, while VAL blocks angiotensin II receptors. This combination promotes vasodilation, natriuresis, and blood pressure reduction, improving cardiac function and controlling hypertension in HFrEF patients [5]. Its use has been shown to be safe in patients with chronic kidney disease (CKD) [6].

Beyond cardiovascular benefits, SAC/VAL may provide renoprotective effects [7,8]. In patients with CKD and HF, ARNi therapy has been shown to slow renal function decline and reduce albuminuria without significantly increasing hyperkalemia risk [8]. These effects are particularly relevant in hemodialysis patients, where preserving residual renal function is critical.

In this context, we present the clinical case of a young hemodialysis patient with hypertension and HOHF associated with a hyperfunctioning AVF treated with SAC/VAL. We discuss the observed clinical effects, exploring the possibility of a synergistic or cumulative therapeutic impact on cardiac function and hypertension, as well as potential implications for renal function.

This case report was previously presented as a communication at the 31st National Congress of Internal Medicine in Portugal, held in Coimbra from May 22 to 25, 2025.

## Case presentation

A 32-year-old female undergoing hemodialysis for five years due to end-stage CKD of multifactorial etiology, likely reflux nephropathy with chronic pyelonephritis secondary to a congenital malformation (spina bifida with neurogenic bladder), presented with refractory hypertension despite optimized triple antihypertensive therapy (nifedipine 60 mg/day, carvedilol 25 mg/day, and methyldopa 1000 mg/day) and dialysis adjustments. She reported exertional dyspnea, peripheral edema, and jugular venous distension.

**Figure 1:**
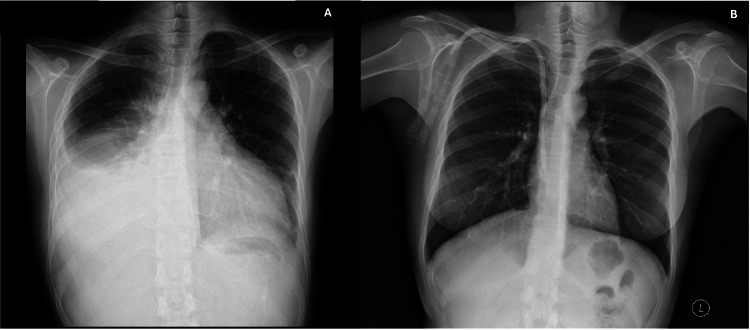
Chest X-ray before and after therapy (A) Posteroanterior chest X-ray before the start of therapy showing recurrent right pleural effusion and increased cardiothoracic index. (B) Posteroanterior chest X-ray six months after initiation of ARNi therapy, demonstrating resolution of pleural effusion and placement of a right jugular vein dialysis catheter as an alternative access following closure of the high-output fistula. ARNi, angiotensin receptor-neprilysin inhibitor

**Figure 2:**
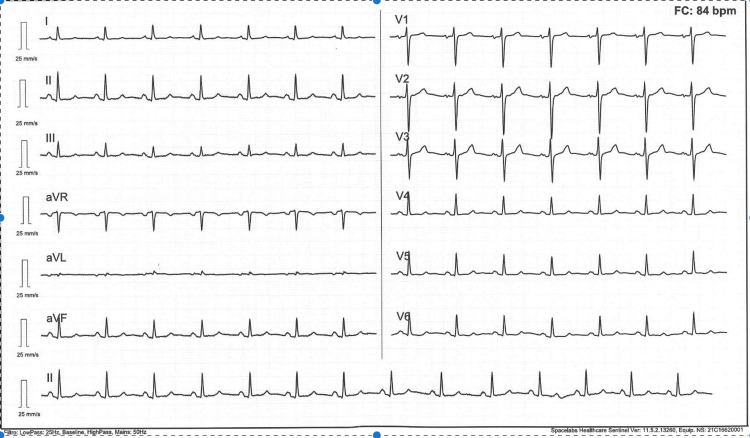
Electrocardiogram The patient’s electrocardiogram was normal, showing sinus rhythm with a heart rate of 84 bpm, a PR interval of 134 ms, and a QTc of 419 ms within normal limits. No abnormalities of ventricular repolarization were observed.

**Figure 3:**
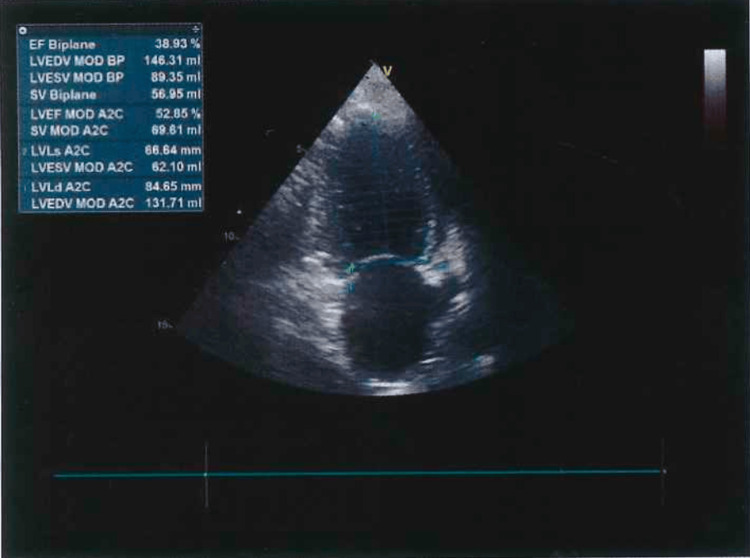
TTE at diagnosis TTE demonstrated reduced LVEF of 38% and PASP of 46 mmHg. LVEF, left ventricular ejection fraction; PASP, pulmonary artery systolic pressure; TTE, transthoracic echocardiography

She was referred for evaluation of recurrent pleural effusion (Figure 1A), despite optimized ultrafiltration during dialysis. Physical examination revealed a high-output AVF (Qa >3000 mL/min), jugular venous distension, and bilateral peripheral edema (++/++++). Thoracentesis yielded a transudative pleural effusion. The electrocardiogram was unremarkable (Figure 2). Transthoracic echocardiography (TTE) demonstrated reduced left ventricular ejection fraction (LVEF) of 38%, pulmonary artery systolic pressure (PASP) of 46 mmHg, and N-terminal pro-BNP (NT-proBNP) of 113,883 pg/mL (Figure 3, Table 1).

**Table 1:** Evolution of TTE parameters and NT-proBNP at diagnosis and after combined therapeutic measures NT-proBNP: Universal rule-out cutoff concentration <300 pg/mL. Rapid rule-in of acute HF uses age-dependent cutoff concentrations: 450 pg/mL for patients <50 years, 900 pg/mL for patients 50-75 years, and 1800 pg/mL for patients >75 years [9,10]. LVEF: HFpEF is defined as symptomatic HF with LVEF ≥50%. HFmrEF is defined as symptomatic HF with LVEF 41-49%. HFrEF is defined as symptomatic HF with LVEF ≤40% [11]. PASP: Values >30 mmHg are considered above the normal range for most healthy individuals [12]. HF, heart failure; HFmrEF, heart failure with mildly reduced ejection fraction; HFpEF, heart failure with preserved ejection fraction; HFrEF, heart failure with reduced ejection fraction; LVEF, left ventricular ejection fraction; NT-proBNP, N-terminal pro-BNP; PASP, pulmonary artery systolic pressure; TTE, transthoracic echocardiography

Evaluation	At diagnosis	After combined therapeutic measures (six months)	Reference values (normal)
NT-proBNP (pg/mL)	113,883	2,005	<300
LVEF (%)	38	71	>50
PASP (mmHg)	46	23	<30

After ruling out other causes of HOHF, such as anemia, thyroid disease, pregnancy, and obesity, the etiology of HF in this young woman was attributed to the presence of a high-output AVF and resistant hypertension.

**Figure 4:**
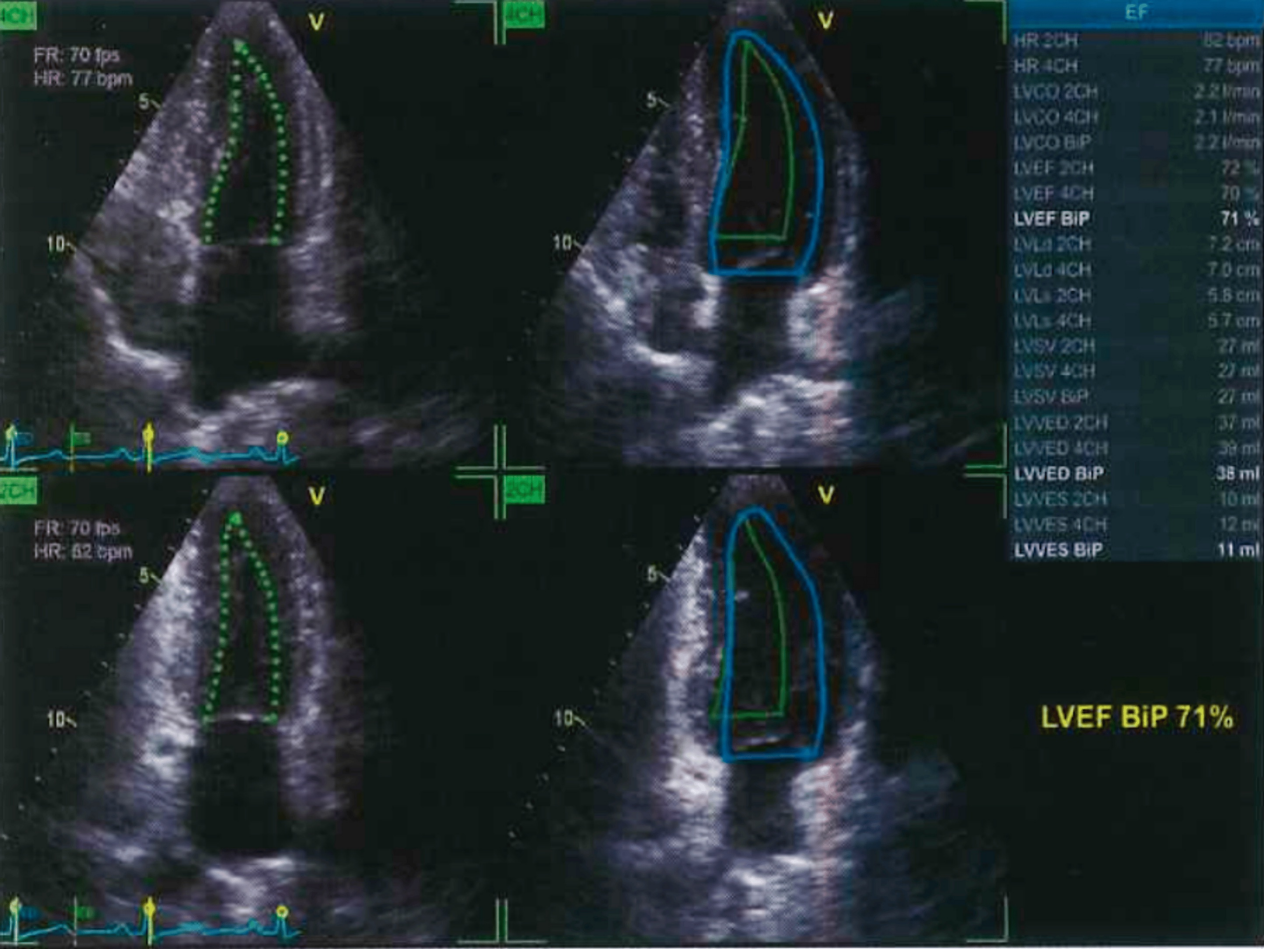
Follow-up TTE six months after initiation of intermediate-dose SAC/VAL and AVF closure, demonstrating recovery of LVEF to 71% and reduction of PASP to 23 mmHg AVF, arteriovenous fistula; LVEF, left ventricular ejection fraction; PASP, pulmonary artery systolic pressure; SAC/VAL, sacubitril/valsartan; TTE, transthoracic echocardiography

All antihypertensive medications were switched to an intermediate dose of SAC/VAL, with close monitoring of serum potassium and blood pressure. Within two weeks, significant improvement in blood pressure and symptoms was observed, along with resolution of the pleural effusion, without reported side effects such as hyperkalemia (potassium >5.5 mEq/L). Two months later, the AVF was closed, and dialysis was continued via a long-term catheter. At six-month follow-up, TTE showed recovery of LVEF to 71%, PASP reduced to 23 mmHg, NT-proBNP decreased to 2005 pg/mL (Figure 4, Table 1), and no pleural effusion was present (Figure 1B). The patient remained clinically stable with no reported adverse effects.

After being diagnosed with HFrEF, the patient was initially removed from the kidney transplant list. Improvement in LVEF with the instituted therapy allowed her to be reinstated, and the transplant was performed two months after reinstatement. Posttransplant, despite complications arising from anatomical alterations due to underlying congenital malformations, the patient is currently dialysis-free, maintaining graft function, but requires a nephrostomy to support renal excretion.

## Discussion

This case highlights the role of ARNi therapy in improving cardiac function and blood pressure control in a young hemodialysis patient with HOHF. The hemodynamic burden imposed by the high-flow AVF also contributed to the development of HF [1,2].

Treatment with SAC/VAL provided early symptomatic improvement, reversed ventricular remodeling, and increased ejection fraction without notable adverse effects. The decision to initiate only a single new agent, with discontinuation of previous antihypertensive medications, was based on the potential for intradialytic hypotension in hemodialysis patients, which may compromise procedure tolerance. As SAC/VAL is a potent blood pressure-lowering drug [4], it could precipitate hemodynamic instability during intermittent dialysis sessions. In this patient, adequate blood pressure control was achieved with an intermediate dose of SAC/VAL alone, and no additional antihypertensive therapy was required. Had satisfactory control not been achieved with the maximum dose of SAC/VAL, the addition of another agent, such as carvedilol, would have been considered to further reduce blood pressure and optimize HF management [4].

In addition to SAC/VAL therapy, the patient underwent AVF closure, which typically reverses HOHF [1,2]. In the authors’ view, these two interventions may have had a synergistic effect on the patient’s clinical and analytical improvement. Furthermore, recovery of cardiac function allowed reinstatement on the kidney transplant list, ultimately leading to successful transplantation.

Although growing evidence supports the benefits of ARNi in HFrEF and its renoprotective effects, data on its use in hemodialysis patients remain limited [4,13-16]. Careful monitoring for potential adverse effects, including hypotension and hyperkalemia, is required. This case contributes to the literature by illustrating a favorable outcome with ARNi in a population typically underrepresented in clinical trials, potentially paving the way for more aggressive ARNi protocols in this setting.

## Conclusions

SAC/VAL was effective and safe for controlling hypertension and improving left ventricular function in a young hemodialysis patient with HOHF secondary to a hyperfunctioning AVF.

This case underscores the importance of a multidisciplinary approach, integrated care, and close monitoring, while highlighting the need for further studies to clarify the role of ARNi therapy in this population. Continuous clinical assessment and individualized management are essential for effectively addressing complex conditions in dialysis patients.
